# Computational Neuroscience Applied in Surface Roughness Fiber Optic Sensor

**DOI:** 10.1515/tnsci-2019-0012

**Published:** 2019-04-23

**Authors:** Wei He

**Affiliations:** 1 School of Electronic Engineering, Xi'an University of Posts and Telecommunications, Xi'an 710121, China

**Keywords:** Computational Neuroscience, Surface roughness, Optical fiber, Sensor, Radial basis function

## Abstract

Computational neuroscience has been widely used in fiber optic sensor signal output. This paper introduces a method for processing the Surface Roughness Fiber Optic Sensor output signals with a radial basis function neural network. The output signal of the sensor and the laser intensity signal as the light source are added to the input of the RBF neural network at the same time, and with the ability of the RBF neural network to approach the non-linear function with arbitrary precision, to achieve the nonlinear compensation of the sensor and reduction of the effect of changes in laser output light intensity at the same time. The Surface Roughness Fiber Optic Sensor adopting this method has low requirements on the stability of the output power of laser, featuring large measuring range, high accuracy, good repeatability, measuring of special surfaces such as minor area, and the bottom surface of holed etc. The measurements were given and various factors that affect the measurement were analyzed and discussed.

## 1 Introduction

Surface roughness is one of the most important parameters for monitoring the machining process and workpiece quality. Although the current profiler can accurately detect surface roughness, it is not suitable for on-line measurement and control in automatic manufacturing, and destructive contact measurement is often not allowed for superfinishing [1, 2]. For this reason, people have been trying to develop non-contact photoelectric methods and devices to measure surface roughness. Although the Intensity Modulated Fiber Optic Sensor (IM-FOS) has the advantages of simple structure and low cost, the drift of the light intensity of light source and some interference light have a great influence on the signal stability of the sensor. If this effect can be effectively eliminated and the measurement signal is sufficiently stable, the application prospect of this kind of sensor will be broad. The Intensity Modulated Fiber Optic Sensor (IM-FOS) will measure the surface roughness based on the principle of scattering, so the measurement is not only related to the processing quality of the workpiece, but also directly related to the cleanliness degree of workpiece surface [3]. Therefore, to measure the surface roughness by the electro-optical method, the cleaning of the workpiece surface must be standard, which is essential to ensure the repeatability of the measurement.

In 1929, the German first quantitatively evaluated the height of the surface microscopic anomalies, and published a monograph on surface roughness, which was proposed in the book. Evaluate the concept of the parameter H max and the measurement baseline. These two concepts are a qualitative leap in the history of surface roughness research, and since then began a quantitative description of surface roughness. The same generation also contributed to the establishment of the measurement baseline. In 1936, EJAbbott developed the first surface roughness instrument for the workshop. This instrument used a relationship between the depth of the peak of the profile and the ratio of the support area, i.e. the Abbott curve to characterize the surface roughness. In 1940, the British successfully developed the Talysurf stylus surface roughness measuring instrument. Since then, countries have also developed a number of contour gauges for measuring surface roughness. In 1951, the Oputon plant in the Federal Republic of Germany produced an interference microscope with surface roughness. In 1978, Taylor-Hobson developed the Talysurf-5 surface profiler. It uses an electronic computer to process the data and directly displays the measurement results for 15 evaluation parameters. In recent years, the emergence of scanning electron microscopy has opened up a new way for the measurement of surface roughness.

With the development of production and the improvement of the technological level, the surface quality of the parts has been raised more and more, especially the development of ultra-precision machining technology and optical technology, making the surface roughness measurement technology develop towards the Nano-level. Countries have started research work in this area. With the continuous development of computer technology, quantitative measurement of surface roughness in numerical situation has become the main means of surface roughness measurement. The long-term production practice shows that: reducing the surface roughness of the parts is of great significance for ensuring reliable and stable cooperation, improving the wear resistance and corrosion resistance of the parts and improving the working precision and sensitivity of the machine and the instrument. Therefore, the research and testing of the surface roughness formation mechanism has become an important research direction.

There are many methods for measuring roughness. At present, the methods for quantitatively evaluating surface roughness are mainly two types of contact measurement and non-contact measurement. Contact measurement is difficult to meet online detection, and also has a certain impact on the surface to be processed and its own device; non-contact measurement speed is fast, can meet the needs of high-speed, automatic, non-destructive testing, and has great potential for on-line detection of roughness.

The remains of this paper are organized into four sections. Section 2 contains working principle of optical fiber displacement sensor. In section 3, we give the principle and composition of neural network. In section 4, we give the training of neural network. In the last section we draw the conclusion over our research.

## 2 Working principle of optical fiber displacement sensor

**Figure 1:**
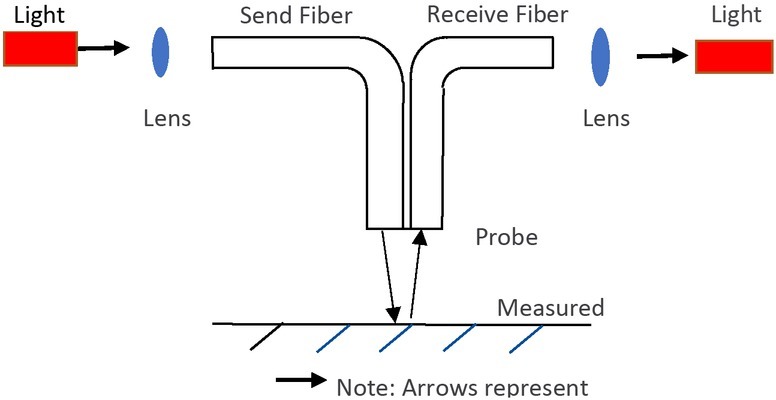
Reflective displacement fiber optic sensor

In the Reflective Fiber Optic Displacement Sensor (Figure 1), the light emitted by the light source is transmitted to the surface (reflection surface) of the measured object through the send fiber, and the reflected light is collected by the receive fiber, and sent to the optical detector to convert into electrical signals for output. The displacement of the object from the probe can be measured via the electrical signal. The light has a certain size of aperture, when the tip of the fiber optic probe is close to the measured object, the light in the send fiber cannot be reflected into the receive fiber, and there is no light signal in the received light. When the measured surface is gradually away from the fiber optic probe, the area for the send fiber illuminating the measured surface becomes larger [4, 5].

**Figure 2:**
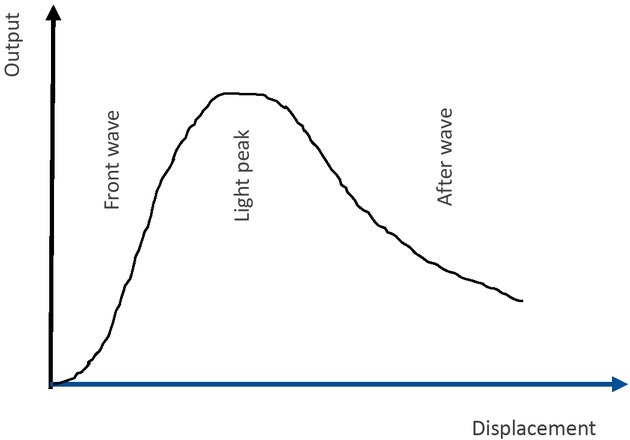
Change in output with displacement

As a result, the illuminated area on the receive fiber end is getting larger, with a linearly increasing output signal. When the entire receive fiber is fully illuminated, the output signal reaches the Light Peak point on the Displacement-Output signal curve (Figure 2).

The curve before the Light Peak point is called the front slope area. When the measured surface continues to move away, some of the reflected light is not reflected into the receive fiber, and since the receive fiber is farther away from the measured surface, the received light intensity gradually decreases, and the

output signal of the photosensitive element gradually weakens, entering the After Wave area of the curve. In the Front Wave area of the Displacement-Output curve, the intensity of the output signal increases quickly. This area can also be used to measure 1 the roughness in micrometers. The attenuation of the signal in the After Wave area is inversely proportional to the square of the distance between the probe and the measured surface, suitable for measurements with large distance and low requirements on sensitivity, linearity and accuracy. In the Light Peak region, the signal reaches the maximum value, depending on the state of the measured surface. Therefore, this region can be used for optical measurement of the surface state, that is, measurement of roughness.

## 3 The principle and composition of neural network

### 3.1 RBF neural network

Neural networks have broad and attractive prospects in system identification, pattern recognition, intelligent control and other fields, especially in intelligent control. People are particularly interested in the self-learning function of neural network, and regard it as one of the keys to solving the problem of controller adaptability in automatic control [6].

Compared with digital computers, artificial neural networks are closer to the human brain in terms of their constituent principles and functional characteristics. They do not perform calculations step by step according to a given procedure, but can adapt themselves to the environment, summarize laws, and accomplish operations, identification or process control [7]. Artificial neural networks must first learn with certain learning guidelines before they can work. For example, the artificial neural network used for displacement is shown in Figure 4.

**Figure 3:**
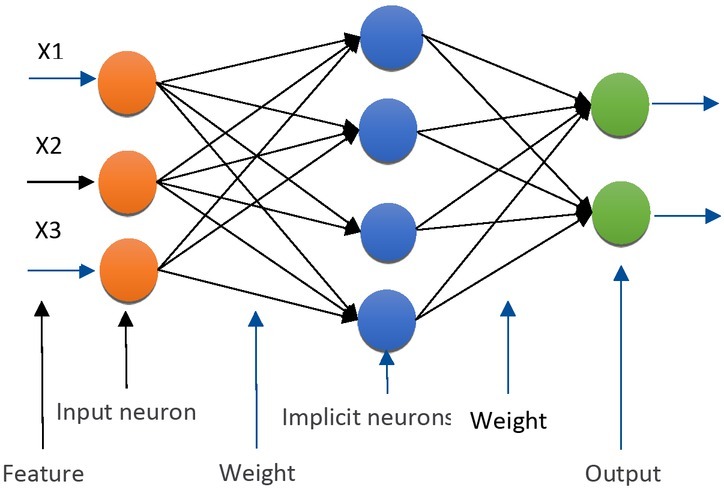
RBF neural network

**Figure 4:**
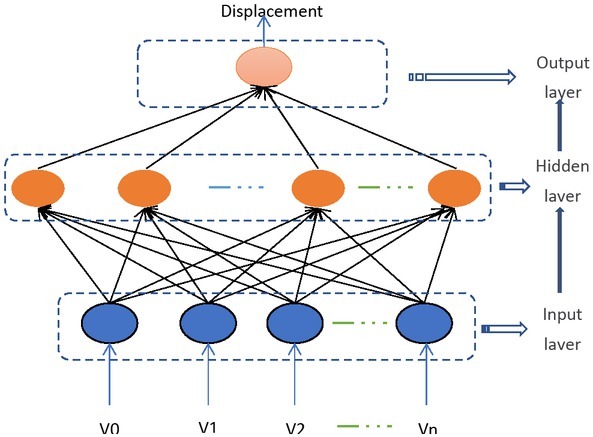
Artificial Neural Network for Displacement

The RBF neural network has stronger approximation ability and faster convergence speed than the BP neural network which is widely used. By training the RBF neural network, it can approximate any nonlinear function, and the learning speed is 103-104 times faster than the BP neural network. The RBF is used as the “base” of the hidden layer unit to form the hidden layer space, so that the input vector is directly mapped (without the weight connection) to the hidden layer space, and the hidden layer space to the output space is linear, and the output of the network is the hidden layer. The linear weights sum of the unit outputs. Therefore, the network’s mapping from input to output is non-linear, while the network output is linear to tunable parameters. In this way, the power of the network can be obtained by directly solving the linear equations, which not only accelerates the training accuracy, but also effectively avoids local minima.

### 3.2 System design

The surface roughness of parts is random. The main reason is that different processing methods lead to different regular geometric contours. When a focused laser beam hits the surface of the object, for the shape of the working surface and the original position of micro area are different, reflection and scattering occurs for the reflected light, and the rate of change of luminous flux is related to measurement. Experiments show that there is a nonlinear relationship between the luminous flux of reflected light and the roughness. Although some person use the Gaussian curve coefficient method to analyze the roughness, but the formula is derived according to the scalar theory, rather than the vector theory, so there is a certain deviation between the theoretical calculation and the measurements. The excellent nonlinear ability, generalization ability and learning ability of neural network can be fully used to solve the problem by avoid finding a precise general data model [8].

For the control target surface roughness), *Y* = *f* (*x*) fals is available. Because the relationship is a kind of nonlinear mapping relationship, and the measure effect of the related parameters is different, while the BP neural network has a non-linear approximation capability and the effect can be expressed using the weight, so the BP neural network is more suitable.

## 4 The training of neural network

The surface roughness of the parts is random; the main reason is that different machining methods lead to different inherent geometric contours. When a focused laser beam is irradiated onto the surface of the object, the difference in surface shape and micro-area position of the workpiece causes specular reflection and scattering of the reflected light, and the rate of change of the luminous flux is related to the roughness. Experiments show that there is a nonlinear relationship between the reflected light and the scattered light flux and roughness. Although some people use Gaussian curve coefficients to analyze the roughness, since the formula is derived from scalar theory rather than vector theory, the theoretical calculation has a certain deviation from the measured results. Making full use of the good nonlinear, generalization and learning ability of the neural network can solve this problem by avoiding the search for accurate general mathematical models.

A well-designed neural network is trained to be able to recognize roughness based on characteristic parameters. The training room acquires characteristic parameters online, and the roughness is taken offline as a sample. The off-line roughness is measured using a Talysufr-4 profiler. Multi-sampled data can be obtained. This sample implements the modified weight of Error Back Propagation for the BP network. After the network error meets the required range for the trained network, the roughness can be obtained based on the forward calculation of the characteristic parameter neural network.

### 4.1 Working principle

The laser emitted from the laser emitter passes the fiber and enters the coupler, and is divided into two paths. One is directly converted into an electric signal by Photodetector 1, and after being amplified and converted into a digital signal *Ui*, it is used as a reference input signal of the RBF neural network. The signal is only related to the characterization laser output intensity *I*. The signal can be expressed as:

(1) Ui=f(IO)

A laser beam from the coupler is sent to a GRIN fiber optic probe via a Y-type optic fiber coupler, and the collimated light is output. After the collimated light is irradiated perpendicularly on the surface of the measured object, part of the reflected light and the scattered light are returned to the GRIN fiber lens. According to the light intensity modulation principle, the luminous flux returning to the GRIN fiber lens is related to the surface roughness of the measured object, the rougher the measured object, the smaller the luminous flux. The light returning to the fiber enters the photodetector, which is amplified and converted into a digital signal *Uri*. As another output signal of RBF neural network, it is found from the analysis that the signal is not only related to the surface roughness of the measured object, but also

reflects the output intensity IO of the laser. The signal can be expressed as:

(2) Uri=g  (Ra,IO)

If the laser output power for the measurement is not stable, then IO in Equation 5 will change, and will also change accordingly. Due to the complex nonlinear relationship between the reflection or scattering light intensity of the object surface and the surface roughness, Equation 2 is actually a nonlinear function that changes with time, directly using *Uri* as a roughness signal, and the error of roughness measurement is large. At present, the above nonlinear function cannot be expressed in an analytical expression. Our goal is to compensate for nonlinearities by the characteristics that RBF neural network can approximate nonlinear functions with arbitrary precision, and introduces a reference input signal *Ui* into the neural network to attenuate change of *Ur* due to change in laser output power, so as to get a signal *U* that is linear with the roughness of the object and truly reflects the roughness of the object.

If Equation 1 is strictly monotonous, and Equation 2 is also strictly monotonic for each IO, and the following equation is got:

(3) Ra=h(Uri,  Ui)

Since Equation 2 is a nonlinear function, Equation 3 must also be non-linear, and it cannot be expressed in analytical form, but it can be approximated by RBF neural network, getting

**Figure 5:**
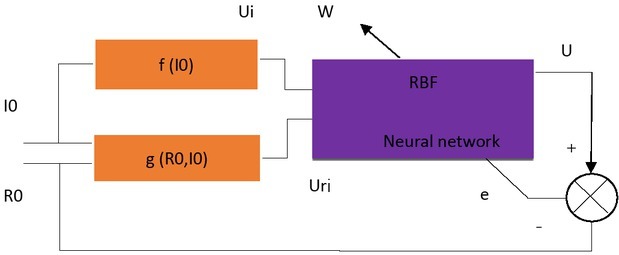
Training of RBF neural networks

(4) U=h(Uri,   Ui)=Ra

so as to achieve non-linear compensation and reduce the impact of laser output power change. To achieve the approximation of Equation 4, the experimental calibration data of the surface roughness fiber optic sensor should be used as a data set for training of the neural network. The training methods are shown in Figure 5. During training, *Uri* and *Ui* are added to the input of the RBF neural network to obtain the actual output *U* of the network. The difference between *U* and the expected number*Ra* is used to adjust the weight *W* of the network, so that *U* constantly approaches *Ra*, getting

(5) (U- Ra)≤X

Where *X* is a given small positive number.

### 4.2 RFB Artificial neural network sample training model

In Table 1, *Ra* is the calibrated surface roughness value, *p* represents the fluctuation of the laser output power, (the presence of *p* will cause change to the light intensity IO), the output of sensor in front of the neural network processor, *U* is the output of the RBF neural network.

**Table 1:** Surface roughness analysis based on RBF neural network

*Ra* Micron	D(-5%)		P(0%)		D(+5%)	
	Uri/V	U/V	Uri/V	U/V	Uri/V	U/V
Q1	0.880	0.100	0.920	0.100	0.970	0.100
Q2	0.480	0.200	0.500	0.200	0.520	0.200
Q3	0.310	0.400	0.320	0.400	0.330	0.400

It can be seen from Table 1 that there is a nonlinear relationship between *Uri* and *Ra*, moreover, with change to the laser output

power, *Uri* will also change and cause error. Table 1 also shows some output data of the sensor after the trained neural network is processed. Comparing that before and after the processing, it can be found that, the surface roughness after processing by the neural network is less affected by the laser output power, and the nonlinearity has been compensated. The neural network has good generalization performance in the training data set range, that is, the network has interpolation characteristics. In order to test the generalization ability of neural network, an untrained set of calibration data is tested, and the result was satisfactory.

### 4.3 BP Artificial neural network sample training model

The GRIN fiber lens is kept perpendicular to the test piece and the distance is 500 microns. It is monitored with an optical power meter so that optical power of 300 microwatts enters the GRIN fiber lens by a semiconductor laser (wavelength of 1300 nanometers). 4 standard blocks are tested 10 times within half an hour. The intensity of reflected light is shown in Table 2. It can be seen from the table that for the same roughness of blocks, the reflected light power measurement has a certain fluctuation, that is, there is a measurement error.

Main causes of error

(1) The fluctuation of the light source is the main cause of measurement error. Although a rigid-stable semiconductor laser with negative feedback is used, the output optical power still has fluctuations of ±1%, directly affecting the measurement of the reflected light power.
(2) The distance between the sensor and the sample block changes, resulting in measurement error. Because of the irregularity of the standard sample block, the distance between the sample block and the sensor is different for the measurement of different points, and the angle of the scattered light measured by the sensor changes. The measured scattered light intensity is also different. Third, the micro-bending and vibration of the transmission fiber will change the light transmission characteristics and affect the measurements.

**Table 2:** Reflected optical power when measuring different roughness samples

Roughness reflected light power measurement times		0.012	0.025	0.050	0.100
	1	6.10	5.80	5.26	4.96
	2	6.15	5.77	5.22	4.93
	3	6.07	5.65	5.29	4.90
	4	6.15	5.65	5.14	4.88
	5	6.08	5.73	5.15	4.86
	6	6.21	5.82	5.17	4.92
	7	6.21	5.80	5.22	4.89
	8	6.16	5.83	5.20	4.91
	9	5.95	5.60	5.33	4.95

To get the test results of the neural network, the experimental data in Table 1 is sent to the neural network for training. The BP algorithm was used to determine the weights after nearly a thousand iterations. The system tested each of the four standard samples 10 times. The results are shown in Table 3. It can be seen from the table that trained neural networks have strong recognition capabilities.

**Table 3:** Neural Network Test Results

Standard specimen roughness neural network calculation value measurement times	0.012	0.025	0.05	0.1
1	0.012000	0.024892	0.049884	0.099743
2	0.011964	0.024942	0.050023	0.099907
3	0.012023	0.025147	0.049782	0.100070
4	0.011964	0.025147	0.050306	0.100180
5	0.012015	0.025009	0.050270	0.100292
6	0.011921	0.024859	0.050119	0.099961
7	0.011956	0.024892	0.050023	0.100126
8	0.011956	0.024843	0.05009	0.100016
9	0.012116	0.025236	0.049646	0.099798
10	0.012116	0.025026	0.050023	0.099907

## 5 Conclusions

In this paper, with the ability of the neural network to approach non-linear functions with arbitrary precision, and the advantages of fast training speed, the output signal of the surface roughness fiber optic sensor and the laser output power signal as the light source are added to the input of the neural network at the same time, to achieve nonlinear compensation of the sensor and reduces the influence caused by the change to the laser output power.

## References

[1] Bazaka K., Jacob M. V. (2012). Implantable devices: issues and challenges. Electronics.

[2] Kinet D., Mégret P., Goossen K. W., Liang Q., Heider D., Caucheteur C. (2014). Fiber bragg grating sensors toward structural health monitoring in composite materials: challenges and solutions. Sensors.

[3] Aksak B., Murphy M. P., Sitti M. (2007). Adhesion of biologically inspired vertical and angled polymer microfiber arrays. Langmuir the Acs Journal of Surfaces & Colloids.

[4] Hou D., Wang J., Sun X., Ji Z., Luan Z. (2012). Preparation and properties of pvdf composite hollow fiber membranes for desalination through direct contact membrane distillation. Journal of Membrane Science.

[5] Zheng G., Cui X., Yang C. (2010). Surface-wave-enabled darkfield aperture for background suppression during weak signal detection. Proceedings of the National Academy of Sciences of the United States of America.

[6] Prajapati C. S., Kushwaha A., Sahay P. P. (2013). Effect of al dopants on the structural, optical and gas sensing properties of spray-deposited zno thin films. Materials Chemistry & Physics.

[7] Pearce S. J., Charlton M. D. B., Hiltunen J., Puustinen J., Lappalainen J., Wilkinson J. S. (2012). Structural characteristics and optical properties of plasma assisted reactive magnetron sputtered dielectric thin films for planar waveguiding applications. Surface & Coatings Technology.

[8] Huang J., Křemenáková D., Militký J., Zhu G., Wang Y. (2015). Evaluation of illumination intensity of plastic optical fibres with.

